# ECMO as a bridge to cardiac surgery: stabilizing unstable patients for a definitive procedure

**DOI:** 10.1007/s12055-023-01523-6

**Published:** 2023-05-22

**Authors:** Jai Raman, Pankaj Saxena, Nikola Dobrilovic

**Affiliations:** 1 Department of Cardiothoracic Surgery, St Vincent’s Hospital, University of Melbourne, Fitzroy, Australia; 2 Department of Cardiothoracic Surgery, Townsville University Hospital, Townsville, QLD Australia; 3 Section of Cardiac Surgery, Evanston Northshore Health System, Chicago, IL USA

**Keywords:** ECMO, Cardiac surgery, Bridging therapy

## Abstract

**Introduction:**

Extracorporeal membrane oxygenation (ECMO) in adults has been used in post-cardiotomy patients who decline hemodynamically. Cardiogenic shock in patients with potential surgically correctable cardiac conditions are at significantly higher risk for post-operative morbidity and mortality. We present experience with a pre-emptive approach of ECMO institution pre-operatively to stabilize patients with cardiogenic shock.

**Materials and methods:**

This study expands on a pilot study with a group of twenty patients who were supported with ECMO pre-operatively in different institutions over a period between 2011 and 2021. The patients presented with cardiogenic shock. Peripheral veno-arterial (VA) ECMO support was used in all the patients. Cardiac surgery was performed via median sternotomy utilizing the in situ ECMO cannulae to institute cardiopulmonary bypass (CPB).

**Results:**

Seventeen patients were weaned off ECMO support following a mean duration of support of 156 h. Fifteen patients survived to discharge. The 30-day mortality and in-hospital mortality were 25% (expected 67% by European System for Cardiac Operative Risk Evaluation (EuroSCORE) II). The causes of mortality included persistent bleeding in 2 patients due to liver dysfunction, and one with low platelet counts. The other two had multi-organ failure.

**Conclusions:**

Variable period of pre-operative ECMO support provides hemodynamic stability and may prevent or reverse the multi-organ dysfunction if instituted on time in patients presenting with cardiogenic shock. This strategy allows cardiac surgery to be performed with acceptable risk.


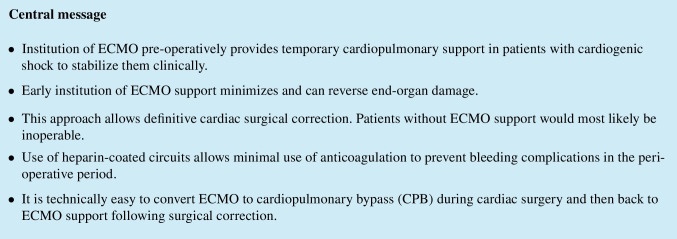


## Clinical vignette

**Fig. 1:**
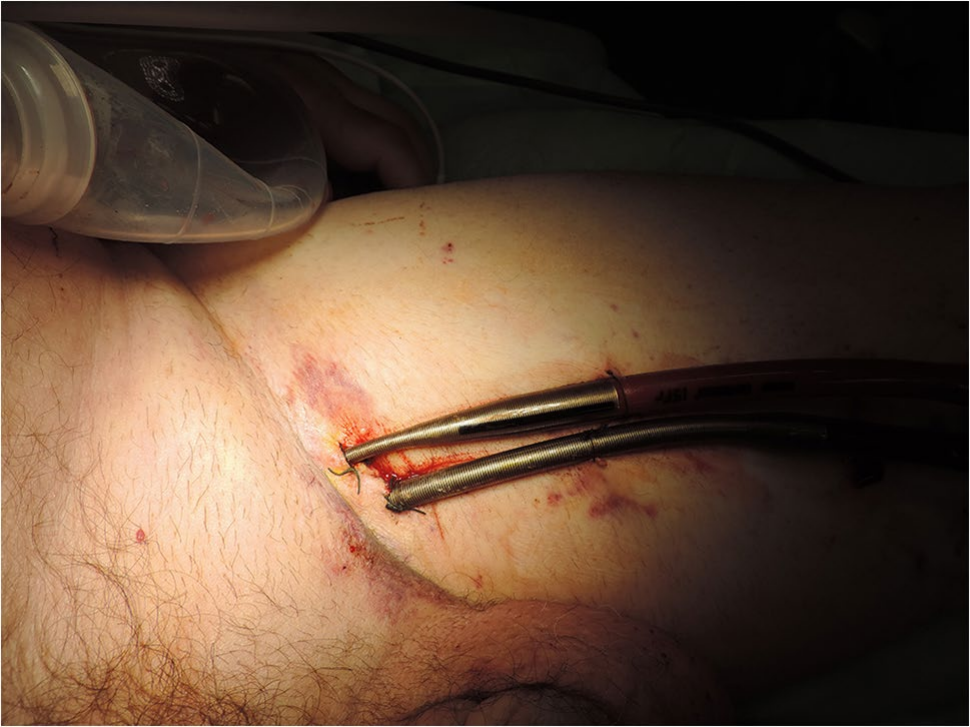
Peripheral cannulation using a 15-Fr Biomedicus arterial and 23-Fr multi-hole Biomedicus venous cannula

A 54-year-old male weighing 82 kg presented with a suspected inferior ST elevation myocardial infarction (STEMI) 1 week after attempted right atrial flutter ablation. The patient was in cardiogenic shock by the time he got to the cardiac catheterization laboratory. The angiogram revealed an acute and abrupt cut-off of the right coronary artery (RCA) at the crux and evidence of an inferior ventricular septal rupture (VSR). The patient had been loaded with clopidogrel and was on dabigatran. He also had a significant 80% stenosis of the proximal left anterior descending (LAD) coronary artery. Although he was still awake, he was decompensating rapidly. After a quick multi-disciplinary discussion, femoral arterial and venous sheaths were placed in the left groin vessels. The patient was taken to the intensive care unit (ICU). Under echocardiographic guidance, a 15-Fr Biomedicus (Medtronic) arterial cannula was inserted through the left femoral artery and the tip positioned in the aorto-iliac bifurcation. Figure 1 shows peripheral cannulation using a 15-Fr Biomedicus arterial and 23-Fr multi-hole Biomedicus venous cannula inserted through the left femoral artery and vein.

**Fig. 2:**
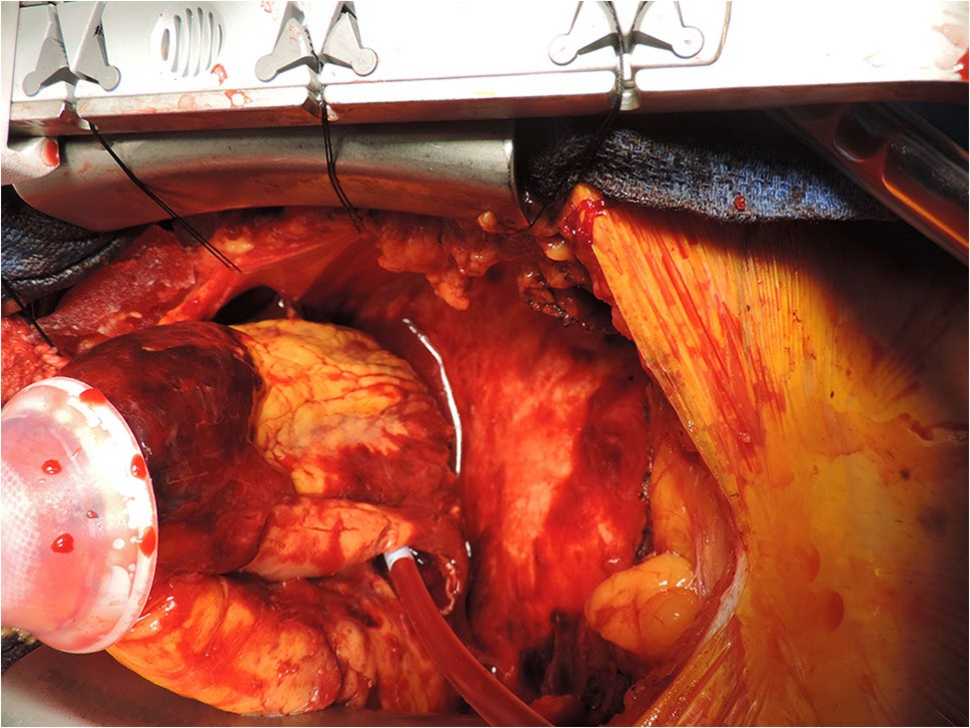
Inferior wall hemorrhagic infarct

A 25-Fr multi-hole Biomedicus venous cannula was inserted through the left femoral vein after appropriate dilatation of the tract over the wire. The patient was then connected to extracorporeal membrane oxygenation (ECMO) using a CentriMag (Thoratec Corporation, Pleasanton, CA) pump with a Quadrox oxygenator (Maquet Cardiovascular AG, Rasstat, Germany) inline. The plan was to wait 3 days of hemodynamic stabilization and for the effects of Plavix and dabigatran to wear off. After 3 days, the patient was taken to the operating room, and a complex procedure performed. The patient was connected to cardiopulmonary bypass using the existing left femoral arterial and venous cannulae; ECMO was paused for the duration of the procedure. There was evidence of a hemorrhagic infarct of the inferior wall of the left ventricle (LV) and right ventricle (RV) with VSR and contained rupture of the inferior wall, illustrated in Fig. 2.

**Fig. 3:**
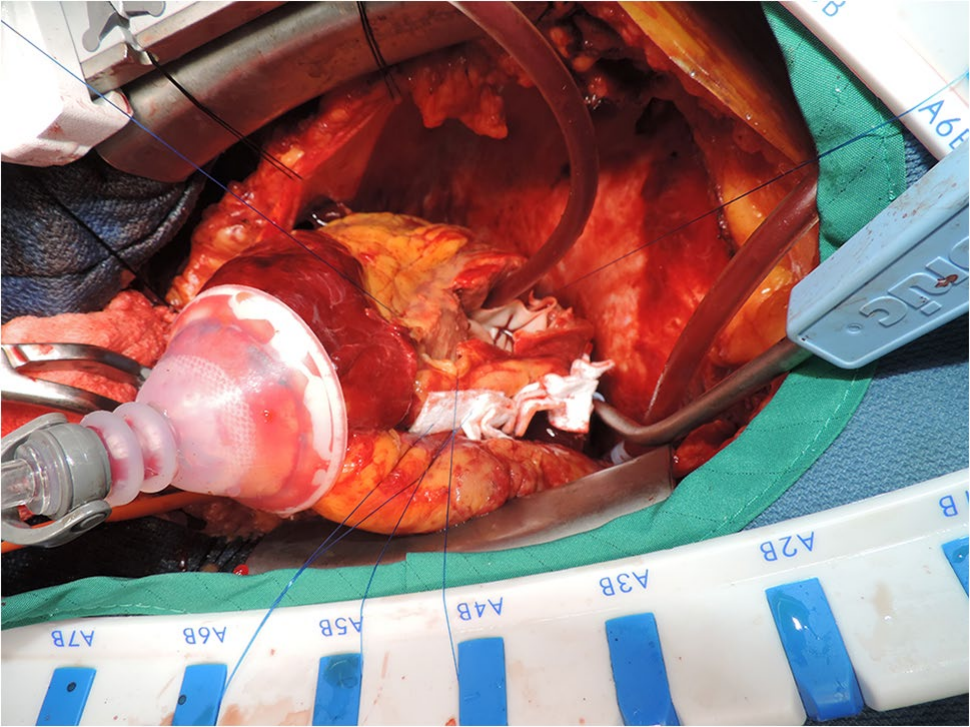
Bovine pericardial patch repair

**Fig. 4:**
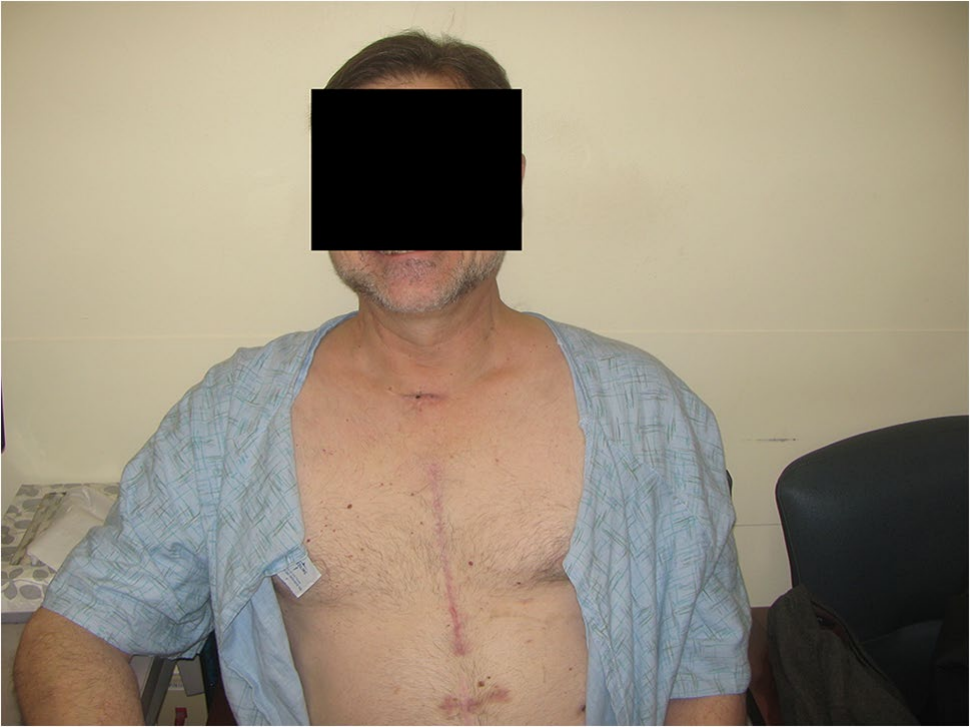
Post-op review at 3 months

The patient underwent a complex reconstruction of the inferior wall and closure of the VSR, using multiple bovine pericardial patches. Figure 3 shows the bovine pericardial patch repair of the ventricles and septal rupture. A left internal mammary artery graft was anastomosed to the LAD coronary artery. The patient was able to be weaned off cardiopulmonary bypass (CPB) uneventfully but was still coagulopathic. This needed multiple infusions of fresh frozen plasma (FFP), cryo-precipitate, platelets, etc. Transfusion of the blood products led to RV dysfunction and hypoxemia. The patient was re-connected to ECMO for the next 4 days and successfully weaned off thereafter. He was transitioned from his endotracheal tube to a tracheostomy on post-operative day 7. This allowed weaning off the ventilator on day 9 and decannulation of his tracheostomy on day 10. He was discharged from hospital 18 days after his admission. On review, 3 and 6 months later, he had recovered fully with a good level of exercise tolerance. Figure 4 shows the patient at his 3 month post-operative review.

This case is being presented and highlighted to illustrate the concept of pre-emptive or prophylactic use of ECMO before the end-organs start failing and as a bridge to conventional cardiac surgery.

## Introduction

Cardiogenic shock causes organ failure due to decreased perfusion. Rapid management of shock can prevent and reverse organ failure. Several cardiac conditions, which are amenable to surgical correction can present with cardiogenic shock. Temporary mechanical circulatory supports (MCS) have found an increasing role in the management of patients undergoing cardiac surgery. Most commonly, the intra-aortic balloon pump (IABP) has been used to support the hemodynamics in patients presenting with acute myocardial ischemia, cardiogenic shock following acute myocardial infarction (MI), mechanical complications of MI such as acute rupture of papillary muscle, or VSR. Balloon pumps are selectively used in patients undergoing cardiac surgery with severely impaired LV systolic function. Traditionally, IABP support has been extensively used in patients who fail to come off CPB support when standard inotropic support is insufficient. However, with improving experience and wider availability of ECMO technology, temporary MCS in the form of ECMO can be provided in a select group of patients who are in cardiogenic shock for better hemodynamic support. ECMO can be used for bi-ventricular failure. It has generally been used as a rescue treatment in patients who have post-cardiotomy shock with or without IABP support. ECMO has also been used in patients who present following cardiogenic shock post-MI, acute myocarditis, acute massive pulmonary embolus (PE), cardiac arrest, etc. Most commonly, ECMO has been used in patients in the setting of peri-operative support in patients undergoing cardiac transplantation or left ventricular assist device (LVAD) [1].

Cardiac surgery in the setting of cardiogenic shock is associated with significant morbidity and mortality. We have utilized ECMO support in patients who present in a hemodynamically unstable and decompensated state as a bridge to decision-making and cardiac surgery. We share our extended experience and provide a protocol-based approach to the use of ECMO support in patients undergoing cardiac surgery [2].

### Inclusion and exclusion criteria

ECMO as a bridge to cardiac surgery is used in patients who are candidates for temporary hemodynamic support to stabilize them and to reverse and prevent the progression of early stage of organ failure. ECMO institution is also indicated in the setting of rising inotrope requirements, poor oxygenation, and worsening acidemia. This strategy is also useful in the retrieval of patients who are in cardiogenic shock at a center that has no provision for cardiac surgery.

Patients with advanced age, frailty, and presence of significant comorbidities and patients who are unlikely to recover cardiac and other organ function are not candidates for ECMO institution. Significant liver dysfunction seems to be a marker of poor prognosis and these patients are not candidates for pre-operative ECMO support [3]. Patients with more than moderate aortic insufficiency (AI) are usually not suitable for ECMO support as a bridging support.

### Decision-making for institution of pre-operative ECMO support

The decision to proceed to pre-operative ECMO support is based on a multi-disciplinary team approach which involves cardiac surgeons, cardiologists, intensivists, and anesthesiologists. Patients presented in their extremis with cardiogenic shock and were deemed extremely high risk or inoperable if taken to the operating room (OR) directly or within the next 24 h. Selected patients appeared to have reversible cardiac function with likelihood of recovery of biochemical and metabolic derangement associated with organ dysfunction. Decision-making involves a family meeting to discuss the high-risk nature of problem and plan of management.

### Technique of cannulation and conduct of ECMO support

**Fig. 5:**
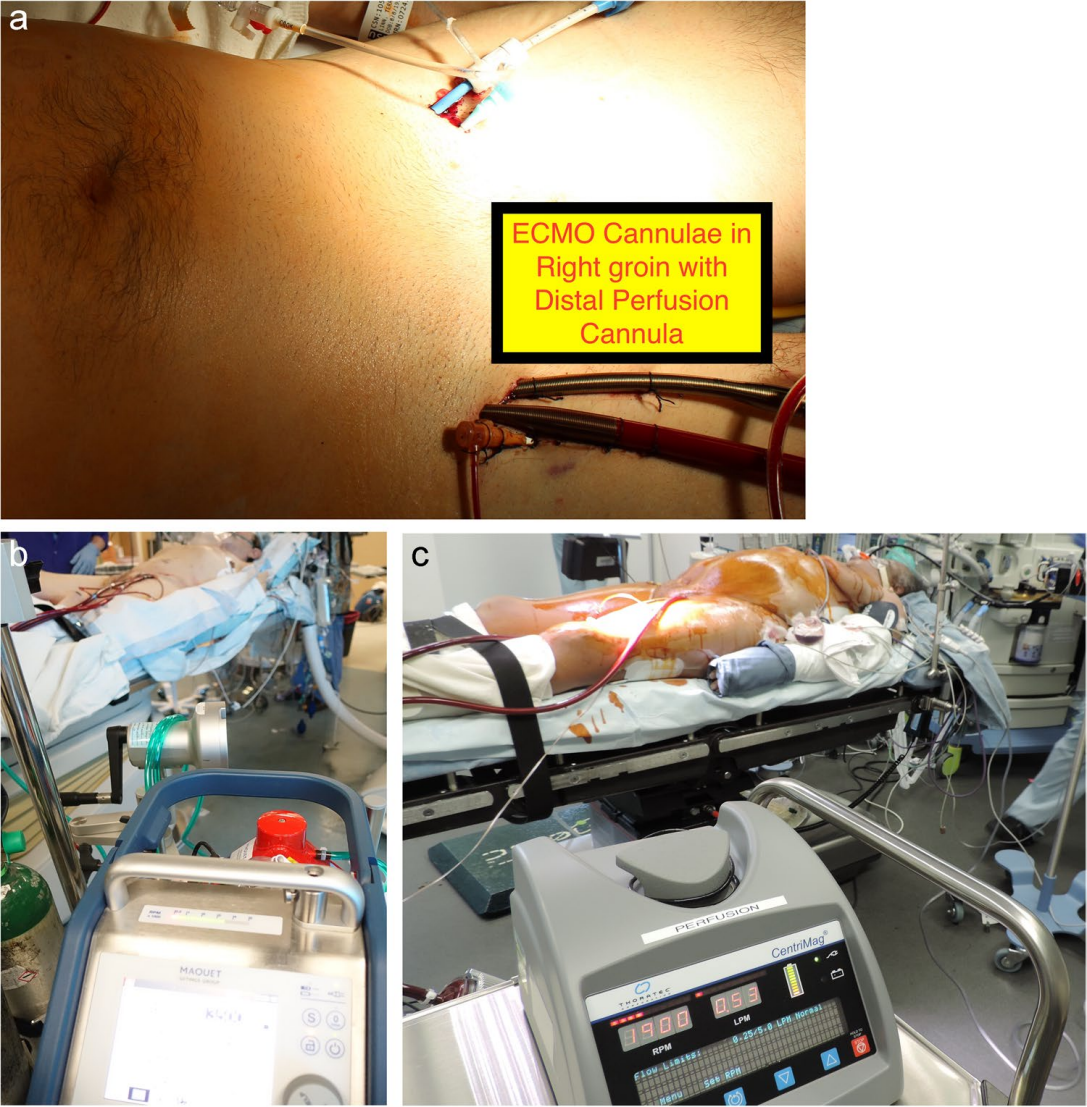
**A** Cannulation of one groin with distal perfusion cannula. **B** ECMO circuit with Maquet CardioHelp system. **C** ECMO circuit with CentriMag system

Usually, veno-arterial (VA) ECMO support is instituted either in ICU or in the OR via femoral access. Anticoagulation is achieved by 5000 U of intravenous unfractionated heparin. Cannulation is usually percutaneous with ultrasound (US) guidance using a Seldinger technique. For arterial cannulation, a 15-, 17-, or 19-Fr Medtronic Biomedicus arterial cannula is used. A distal perfusion cannula with 6- or 7-Fr sheath is routinely used in all cases to prevent distal limb ischemia. This cannula is also placed under US guidance and is connected to a side arm from the inflow cannula. We tend to use a 23- or 25-Fr Biomedicus cannula placed under transesophageal echocardiography (TEE) guidance with the tip of the cannula at the right atrial superior vena caval junction. Cannulae can be placed on the same side of the groin. The circuit is connected to a standard 3/8-in. line circuit. In this experience, we used either the CentriMag system or the Maquet CardioHelp system for ECMO support (Fig. 5 shows cannulation and the circuit configurations). Figure 5(A) shows cannulation of one groin with a distal perfusion cannula to allow blood flow to that lower limb. Figure 5(B) shows an ECMO circuit with the Maquet CardioHelp system. Figure 5(C) depicts an ECMO circuit with the Abbott CentriMag system.

**Fig. 6:**
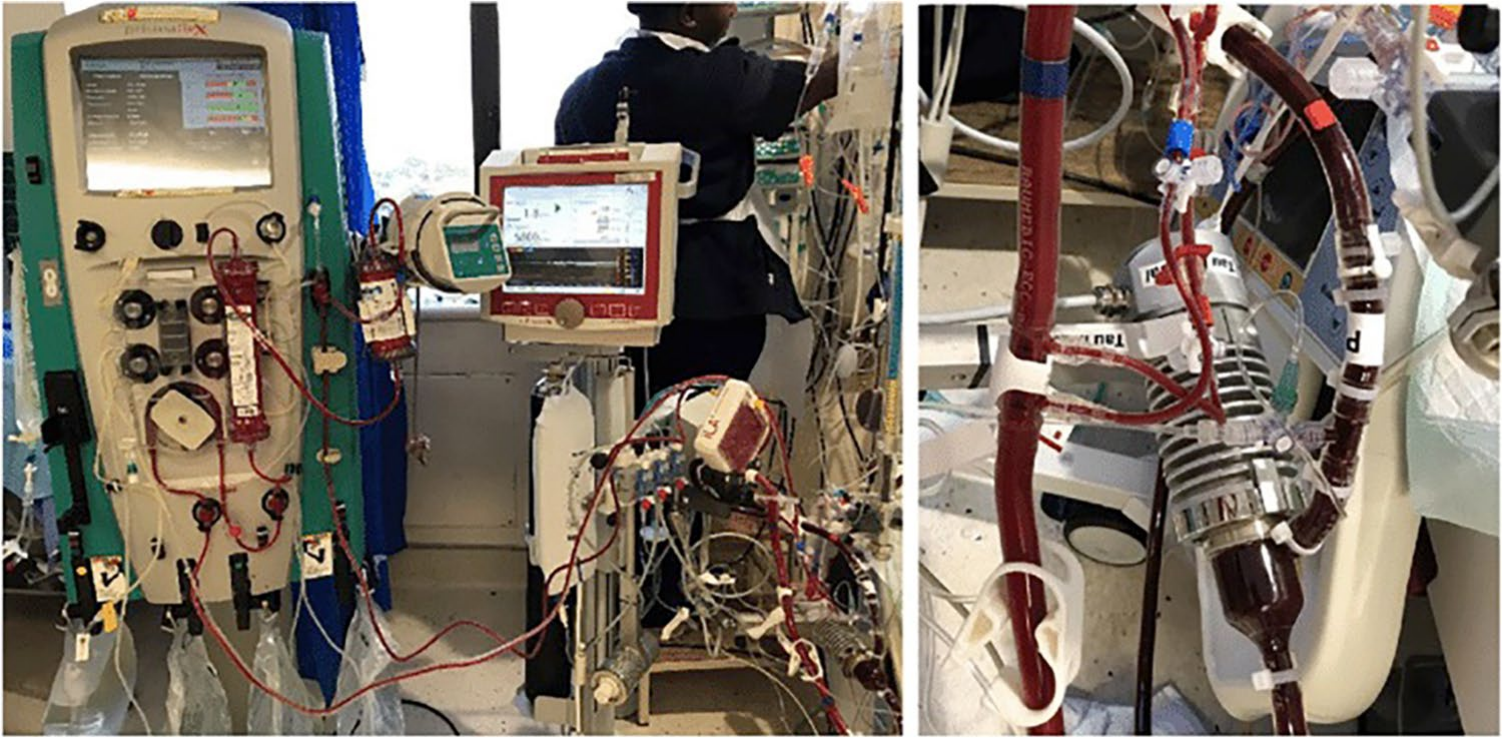
Addition of hemofiltration line to the ECMO circuit

In patients who have difficult femoral vascular access, a semi-open technique is used with cannula placement under direct vision with purse string sutures and with the cannulae tunneled through the skin. This method is especially useful in patients who are morbidly obese or patients who have peripheral vascular disease. A selective approach with simultaneous IABP placement in the contralateral groin can be used to maintain pulsatile flow and wean patients off inotropic support post-operatively. IABP may have already been placed by the cardiology team on admission. Continuous veno-venous hemofiltration (CVVH) or continuous renal replacement therapy (CRRT) can be used for offloading following institution of ECMO. A mini-circuit to hemofilter by ECMO circuit is also available (Fig. 6). Alternatively, this can be achieved by a separately placed vascular access catheter for hemofiltration and/or hemodialysis (VasCath) into a large bore vein.

ECMO flows are maintained preferably at 2–3.5 L/min in patients to allow ejection and flow through the aortic valve. Minimal mechanical ventilatory support is continued during ECMO support to allow native ventilation. Usually, a rate of 10/min and positive end expiratory pressure of 10–15 cm of H_2_O is used. Most of these cases were managed with no additional heparin. Additional anticoagulation is used when flows are low (below 3 l/min) and maintained with intravenous heparin maintaining target activated clotting time (ACT) of 180–220 s.

Cardiac and other organ recovery is assessed by the presence of pulsatile flow, decreasing inotropic requirement, improved renal and liver functions, and improved lactate levels. TEE is utilized frequently to assess the cardiac function while reducing the ECMO flows to allow higher ejection of stroke volume. Echo monitoring is also useful in patients to exclude LV distention that may need to be managed with decompression either percutaneously via trans-septal approach or via thoracotomy.

Generally, a clinical decision can be made after 48–72 h to decide on the timing of surgery.

The median sternotomy approach is generally used for cardiac access. In the OR, the existing cannulae are used for conversion of ECMO support to CPB. Standard heparinization is used during cardiac surgery. The patient may be weaned off ECMO support at the conclusion of the procedure if cardiac function looks satisfactory. However, it may be preferable to transition back to ECMO for temporary support at the end of the procedure if there are any ongoing concerns about the recovery of cardiac function. This strategy also allows hemodynamic management in post-operative period in case of temporary deterioration. We have a lower threshold to leave chest open if hemostasis and myocardial edema are of concern. There is no need to anticoagulate the patients post-operatively as the circuits are heparin-coated, avoiding post-operative bleeding problems. Patients are weaned off ECMO support over next 24–48 h. Decannulation and/or chest closure are done in the OR.

## Results

The biggest published series of pre-emptive ECMO as a bridge to conventional surgery [2] showed a reduction in expected mortality in a very sick group of patients. Since then, the experience has expanded to over 20 patients with expansion into the use of ECMO in patients undergoing trans-catheter aortic valve replacement (TAVR) [4], mitral valve (MV) intervention due to papillary muscle rupture [2], acute RV outflow tract obstruction and failure due to pseudoaneurysm from a previous Bentall procedure [1], acute heart failure decompensation [2] as a bridge to transplantation, and LVAD [2]. Table 1 outlines the details of the various patients.

**Table 1:** Outline of cases with details and outcomes

Age/gender	Operative indications, preoperative diagnoses, and conditions necessitating the bridge approach*	Definitive operation	Hours pre-op	Hours post-op	EuroSCORE II	Hospital mortality	30-day mortality	Complications
(1) 56 M	STEMI, LAD occluded during PCI, cardiac arrest, lactate 6.2, AST 463, ALT 265, RF, creatinine 3.3, pH 7.24, diabetes, HTN	CABG × 4	66	50	64%	●	●	Severe hemoptysis
(2) 52 M	thrombosed P-MV, cardiogenic shock, renal failure, pH 6.93, liver failure, AST 6142, ALT 3567, troponin I 10.64, lactate 12.7, s/p previous congenitally corrected transposition of the great vessels w/MVR, s/p partial MV thrombosis and laparotomy for acute abdomen	Redo-MVR	120	0	87%			AKA
(3) 60 F	STEMI, ostial LM occlusion, cardiac arrest, troponin I > 450, PCI, shock, pH 7.10, lactate 5.6, multiple cardioversions, persistent ventricular arrhythmia, cardiomegaly, Impella, Prasugrel, AST 1136, ALT 336	LVAD	117	0	82%	●		LUE compartment, massive LGIB
(4) 46 F	AV-MV endocarditis, IVDA, MSOF, intubated, cardiogenic shock, renal failure, shock liver, multiple inotropes, lactate 10.5, AST 12,234, ALT 3180	AVR, mitral repair	162	170 (VV)	65%	●	●	UGIB & LGIB
(5) 59 M	STEMI, RCA occlusion, failed PCI, inferior-PVSD, cardiogenic shock, IABP, sepsis, pneumonia, obesity, HTN, sarcoidosis, lactate 8.9	CABG × 1, VSD repair	57	140	89%			
(6) 54 F	Thrombosed P-MV/critical MS, mod-severe AI, markedly dilated right ventricle, CHF, MOSF, intubated, hyponatremia, shock liver, pulmonary edema, pulmonary HTN, DVT, COPD, coumadin, INR 5.4, AST 3655, ALT 3809, Cr 4.4	Redo-MVR, AVR, TV ring	85	290	80%	●	●	
(7) 58 F	STEMI, occluded LAD, occluded RCA, ventricular fibrillation, cardiac arrest, cardiogenic shock, acute respiratory failure, pH 7.16, AST 486, troponin I 218, lactate 9	CABG × 4	210	0	70%			
(8) 51 M	MV & TV *Strep. pneumoniae* endocarditis, severe MR, pneumonia, sepsis, bilateral empyema, pulmonary edema, septic/cardiogenic shock, right pneumothorax, HTN, DM, albumin 1.1	MVR, TV repair, bilateral lung decortication	117	69	75%			
(9) 77 F	Acute MR—chordal rupture, moderate AS, atrial fibrillation, TR, LV dysfunction, pulmonary hypertension, ESRD, s/p renal transplant × 2, NASH, pH 6.97	MV repair	86	24 + 25	69%			
(10) 57 F	STEMI, cardiogenic shock, renal failure, pulmonary hypertension, DM (A1C = 12)	LVAD	355	0	87%			
(11) 61 F	STEMI, occluded large circumflex, papillary muscle rupture, severe MR, pulmonary edema, PHTN, RF, lactate 10, shock, hypotensive even with Impella	MVR, CABG × 1	91	22	81%			
(12) 39 M	Failed BiV pacer upgrade, VT, cardiac arrest, cardiogenic shock, PFO, RF, lactate 12, anuric, Cr > 3, IABP, transfer from OSH	LVAD, PFO closure	88	52	79%			L HTX
(13) 38 M	Previous Ross procedure conversion to Bentall & RV to PA Conduit 14 months earlier, RV failure due to large pseudo-aneurysm arising close to left main coronary button	Redo, repair pseudoaneurysm, repair RV-PA conduit	72	48	68%			
(14) 62 M	Acute MI with ruptured papillary muscle with severe MR & CAD. Impella 5.0 in place	CABG, MVR	56	48	58%			
(15) 74 M	Critical AS with bicuspid AV & mild AR, poor LV function	TAVR	26	12	47%			
(16) 59 M	Acute AMI, cardiogenic shock, Impella 5.0 placed for support initially	Heart transplant	96	0	54%			
(17) 72 M	Critical AS with cardiogenic shock and failing RV	TAVR	48	12	51%			
(18) 60 F	Cardiogenic shock, dilated cardiomyopathy, indwelling Impella 5.0 with hemolysis	LVAD implant	12	12	33%			Renal failure, CVVH
(19) 65 M	Cardiogenic shock, Impella in place	LVAD implant	24	0	44%			Bleeding, re-exploration
(20) 54 M	STEMI with cardiac arrest & cardiogenic shock. Urgent left main PCI	LVAD	96	12				Vent-related issues

^*^Presented data are representative of each patient’s status as encountered by the surgical team at the time of initial consultation, specifically lab values such as troponin I

The protocol of managing anti-coagulation during ECMO, subsequent CPB, and the consequent ECMO support were worked out over the course of the experience. The cannulae used for ECMO support can be used for CPB. While ECMO is maintained with very little or no extra heparin, when CPB is used, this is with conventional full dose heparinization. At the conclusion of the CPB run, heparin is fully reversed with protamine. If further support with ECMO is required, the same cannulae are used with a new ECMO circuit and 5000 units of heparin used to help initiate support.

One patient had an Impella 2.5 inserted through the groin as an initial support for cardiogenic shock. This patient was transitioned to ECMO within 4 h due to rising lactate and worsening cardiogenic shock. Two patients had pre-emptive LV support and then “venting” with an Impella 5.0 device, both of which were removed while the patients were stabilized on ECMO. Neither of them seemed to benefit from the Impella acting as a vent. One patient later in our experience had very poor LV contractility after being salvaged from a large anterior MI. A LV vent was placed through the apex through a mini-thoracotomy. However, this vent catheter had to be manipulated on multiple occasions and pulled back since it had migrated into the left atrium. In summary, 4 of these 20 patients had vents placed in the LV with mixed results and ineffective drainage.

Our predicted mortality based on European System for Cardiac Operative Risk Evaluation (EuroSCORE) II was 67% and actual mortality was 16%, as graphically presented in Table 2.

**Table 2:**
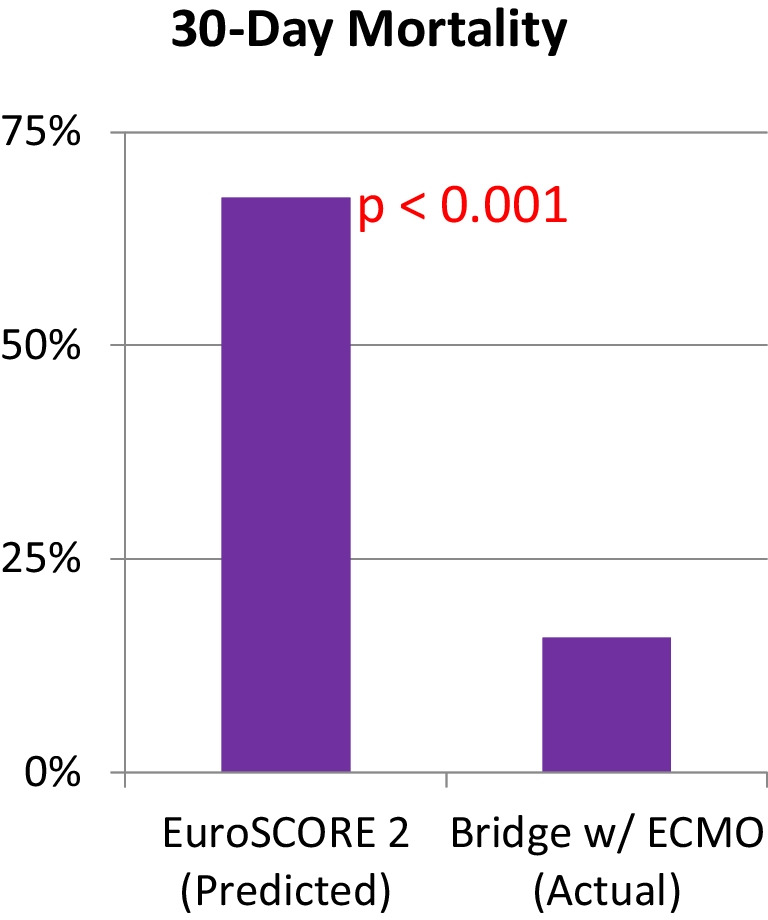
Actual versus predicted mortality based on EuroSCORE II. Mortality outcomes—observed versus expected

### Limitations of the study

This is a retrospective review of clinical data in a sick group and comorbid group of patients. The limitations are that of any retrospective study. No inferences can be drawn on the conduct of the procedures, timing of various interventions, or the judgement exercised by the clinicians involved when confronted with critically ill patients in near-death situations.

## Discussion

ECMO has been used in a wide spectrum of clinical situations with end-stage cardiac disease including bridge to recovery, bridge to bridge, and bridge to cardiac transplant for primary graft failure in patients following transplant. ECMO support has not been utilized to the same degree in non-transplant setting. ECMO support has been utilized in post-cardiotomy setting to wean the patients off CPB support [4, 5]. With increasing clinical experience of ECMO support, there has been wider application of this technology to support patients post-cardiac surgery.

We have found that pre-operative ECMO has the potential to make inoperable patients candidates for cardiac surgery [2]. The mean EuroSCORE II was 67% in 19 patients reported by us previously, representing very high risk associated with surgical intervention. The mean duration of ECMO support was 200 h, with 30-day mortality of 16% mortality. This approach also helps triage the clinical situation in case the patient’s organ function does not improve despite pre-operative ECMO support. Cardiac surgery would be futile in these patients.

This approach has been used in patients awaiting cardiac transplantation as a bridge while their hemodynamics cannot be supported with standard measures. However, the bridge to more conventional procedures almost seems one too far! We have presented our strategy to manage patients requiring non-transplant cardiac surgery and transplantation in cardiogenic shock would have been deemed inoperable. This approach reflects a paradigm shift in salvaging a subgroup of critically ill patients needing cardiac surgery.

LV venting was evaluated in each case and a strategy was utilized based on the underlying cardiac function. Two patients had pre-emptive LV “venting” with an Impella 5.0 device, both of which were removed while the patient was stable on ECMO. The third patient had a vent catheter inserted through the LV apex approached through a mini-thoracotomy. This required manipulation due to migration of the catheter and ineffective drainage. This series of 20 patients therefore is not a good advertisement for LV venting, either with or without the Impella device. Would we have considered pre-emptive support with Impella in any of these patients? We did use it as a first line in 2 patients. One other patient had an Impella inserted beforehand at the referring hospital. Indeed, we were the first group to use Impella through a graft sutured to the right subclavian artery in the USA in 2008, as a stand-alone therapy. This helped facilitate expansion to Impella 5.0 and then 5.5.

However, there has been no consensus on the pre-operative institution of ECMO support in patients with cardiogenic shock. This is only considered class IIB recommendation as per the guidelines released recently in a joint statement by multiple societies [6]. According to the expert consensus, “preoperative implant of ECMO may be considered in patients in very poor condition (hemodynamic or metabolic) or with structural cardiac anomalies (post-MI VSR, severe pulmonary edema or dysfunction due to underlying cardiac disease) to facilitate perioperative management (bridge to surgery)”. It seems that ECMO support is blamed on the poor outcome in patients who undergo surgery in the setting of cardiogenic shock. It needs to be highlighted that these patients would otherwise not survive if no MCS support is used. It is important to emphasize that timing of ECMO institution pre-operatively is paramount. The outcome can be dependent on early use of ECMO support before irreversible organ dysfunction appears, making use of ECMO and surgery futile [7].

Peripheral cannulation was the preferred option since this was easy to deploy in a fairly expeditious manner by the bedside. Central cannulation was not considered since this would have required a trip to the OR, obligatory blood loss from the sternal edges, and the need for careful tunnelling of the arterial and venous cannulae to prevent migration and movement during patient transfer.

All of these patients were in established cardiogenic shock (as evidenced by low cardiac index and rising inotrope requirements and serum lactate levels) and IABP support was inadequate. Augmentation of cardiac output in patients with cardiogenic shock can only be achieved to a limited extent with IABP in conjunction with inotropic support. The decision to institute ECMO rather than IABP was based on severity of cardiogenic shock and the judgement that support with IABP on its own was clearly not enough. Four of the patients had indwelling Impella pumps, and five others had balloon pumps inserted prior to institution of ECMO. The primary indication for pre-operative ECMO institution was cardiogenic shock to prevent organ dysfunction, which is a common secondary problem. The indications in all cases were cardiogenic shock as evidenced by a low cardiac output state, rising inotrope use, increasing levels of serum lactate, worsening acidemia, and in some instances failure of mechanical support devices.

Another strategy in these patients is to add IABP support concomitantly to allow weaning of patients from ECMO to inotropic and IABP support. Counter pulsation with IABP allows to maintain pulsatile flow with potential beneficial effect on microcirculation, allowing better organ function [8]. Additional IABP support may provide better coronary perfusion in patients who have undergone coronary artery bypass surgery by improving the flow through the grafts [9]. There is a possibility that concomitant IABP support might help by improving LV decompression [10] in some patients. The effect of combined IABP and ECMO on cerebral circulation is unpredictable depending on the presence of LV stunning. The effects of IABP on neurological outcomes if any is not clearly known [11]. A retrospective review found that concomitant use of IABP and ECMO may allow better weaning off ECMO support post-cardiotomy but does not improve mortality [12]. Clearly, the strategy to use both the devices at the same time should be individualized.

The addition of ECMO therapy can add significantly to the cost of treatment and may not be applicable to all the healthcare systems. However, this is significantly less expensive than use of a ventricular assist device or implanting mechanical support after the surgical procedure. ECMO is also significantly less expensive that Impella implants.

To conclude, even though case numbers remain limited, accumulating data from multiple centers continue to strongly support the beneficial role of ECMO as a salvage tool.

We suggest an approach for proactive use of ECMO as bridge to cardiac surgery to stabilize patients leading up to their assessment of candidacy for a definitive surgical procedure. We can conclude from our limited experience that ECMO allows definitive surgical procedure by treating shock, while stabilizing and improving organ function.

Further studies would be required to better define the role of this proposed strategy to wider groups of patients presenting with cardiogenic shock.

## Data Availability

Data is available from the individual institutions through locally maintained ECMO registries.
